# A commonly overlooked motor neuron disease mimicker

**DOI:** 10.1530/EDM-14-0085

**Published:** 2014-11-01

**Authors:** Manas Ghosh, Ambarish Bhattacharya, Kaushik Ghosh, Atri Chatterjee, Sisir Chakraborty, Sanat Kumar Jatua

**Affiliations:** Sanjiban Hospital, Kolkata, India; 1 Department of Medicine, Sanjiban Hospital, Kolkata, India; 2 Department of Medicine, Malda Medical College, Malda, West Bengal, 732101, India; 3 Department of Neurology, NRS Medical College, Kolkata, India; 4 Department of Medicine, College of Medicine and Sagore Dutta Hospital, Kolkata, India; 5 Department of Medicine, NRS Medical College, Kolkata, India

## Abstract

Motor neuron disease (MND) is a progressive devastating neurodegenerative disease, which universally progresses towards death. Hence, every attempt should be made to find out if there are any treatable conditions, which can mimic MND. Herein, we describe a case of hypercalcaemia due to primary hyperparathyroidism confused as MND and subsequently cured with parathyroid surgery.

**Learning points:**

- Any patient with neurological disorder should have a screening of all the common electrolytes including calcium as electrolyte imbalance can present with paralysis (e.g. hypokalaemia) to amyotrophic lateral sclerosis (e.g. hypercalcaemia).
- No patient should be stamped as having MND without having a proper work-up of all its differentials as there might be a treatable condition masquerading as MND.

## Background

Parathyroid dysfunction, both over-activity and under-activity, leading to disorders in calcium metabolism commonly present with varied neurological manifestations, which can range from deafness to extra-pyramidal symptoms (1) (2). As such, parathyroid is often referred to as the ‘Pandora's box’ of neurology. Hyperparathyroidism commonly presents with easy fatigability and muscle weakness with preserved reflex, which commonly mimics the predominantly lower motor neuron type of spinal muscle atrophy and amyotrophic lateral sclerosis (ALS). ALS is the most common form of motor neuron disease (MND). Till date, there has been no curative therapy for this neurodegenerative disease. Although there have been some reports suggesting that MND and primary hyperparathyroidism (PHP) might be interlinked, successful removal of parathyroid in these patients did not alter their clinical course. Hence, every attempt should be made to search for any treatable cause before finalising the diagnosis of MND.

## Case presentation

A 36-year-old male presented with a 6-month history of generalised weakness and wasting of all four limbs. The wasting involved both distal and proximal parts of the limbs without any fasciculation. There was no cranial nerve involvement, sensory impairment or autonomic nervous system involvement. Past and personal histories were non-contributory. He is a non-vegetarian. He worked in a rice mill and there was no history of exposure to toxic or heavy metals, nor any history of exposure to repeated electric shock. There was no history of unprotected sexual intercourse. There was no suggestive family history. There was no history of alcohol excess or illicit drug usage.

On examination, he is of average built and nutrition but having significant wasting in both upper and lower limbs. Other parts of general examination were normal. Neurological examination revealed normal higher mental function with normal cranium and spine. Cranial nerve examinations revealed no abnormality. There was no wasting or abnormal movement in the tongue. Examination of the limbs showed more pronounced wasting in proximal muscles and minimum wasting distally. Assessment of power of the limbs showed right upper limb 4/5, right lower limb 4(−)/5, left upper limb 4/5 and left lower limb 4(−)/5, and there was only mild weakness in the small muscles of hands and feet. Tone in all the four limbs was increased but coordination was normal. There was no fasciculation in any muscle group. All the deep reflexes were brisk and superficial reflexes were normal with bilateral flexor plantar response. Examination of his sensory system, extra-pyramidal system and autonomic system (bed side tests) showed no abnormality. He had waddling gait. Examinations of other systems were within the normal limit.

## Investigation

His haemogram was normal except for mild anaemia (Hb 9.8 gm/dl, TLC 11 100/cc [N-65, L-31, M-2, E-2, B-0], ESR-70, platelet – 290 000/dl). Renal function tests were normal (urea 51 mg/dl and creatinine 1.1 mg/dl). FBS was 98 mg/dl and HbA1c 5.8%. Thyroid function was also normal (thyroid-stimulating hormone [TSH] 7.97 mIU/l, FT4 1.1 ng/dl and FT3 3.4 pg/ml). Liver function tests were normal.

Electromyography (EMG) and nerve conduction (NCV) velocity testing of all the four limbs including tongue and paraspinal muscles showed no resting activities (fibrillation/fasciculation) with normal amplitude, latency and recruitment without any conduction block. This ruled out MND. However, due to the presence of classical clinical features, MND mimickers were actively investigated for.

CT scan of brain and cervical region, dynamic X-ray of cervical spine and chest radiograph showed no abnormality. Measurement of serum electrolytes showed Na^+^ 136 mmol/l, K^+^ 4.5 mmol/l, calcium 17.9 mg/dl (normal range 8.8–10.5) and phosphate 3.2 mg/dl (normal range 2.5– 4.9), and his serum parathyroid level (iPTH) was 1541 pg/ml (normal range 15–65 pg/ml). His blood gas analysis showed: PaO_2_ 88 mmHg, PaCO_2_ 36 mmHg, pH 7.38, HCO_3_ 21.4 mmol/l. Viral serology (HbsAg, HIV I and II, and anti-HCV) were non-reactive. ECG showed sinus rhythm at the rate of 78/min, QTc of 391 ms and PR of 152 ms. Toxicological screening of urine was not done because of the lack of history of exposure to chemicals. Serum protein electrophoresis had no evidence of paraproteinaemia. He had normal vitamin B12 (215 pmol/l) and folic acid (25 nmol/l) level. Routine urine examination showed pus cell 34–40/HPF, RBC 0–2/HPF, epithelial cells 0–2/HPF, protein+ and a specific gravity of 1.010.

**Figure 1:**
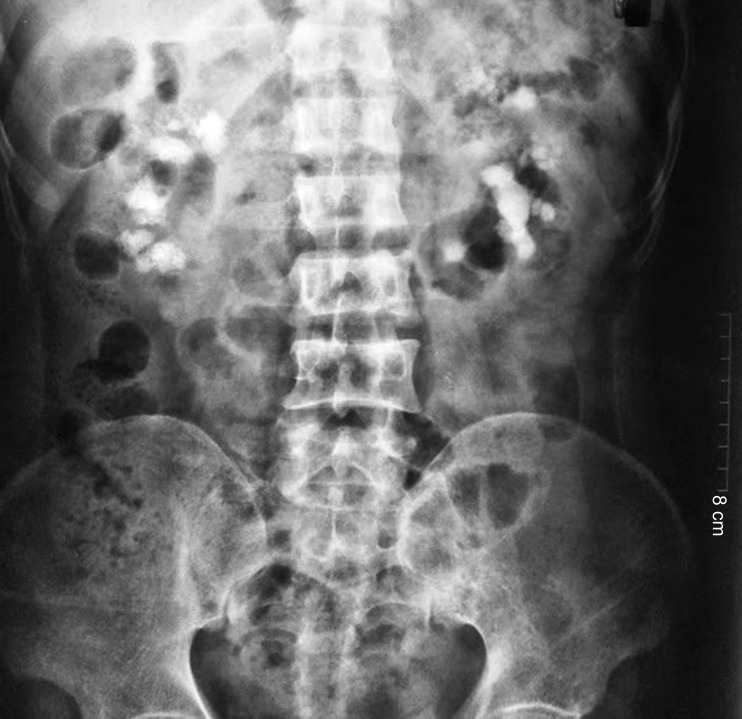
Straight X-ray abdomen showing bilateral staghorn renal calculus without any hydronephrotic changes.

Abdominal ultrasonography and abdominal X-ray revealed bilateral large renal calculi without any hydronephrotic change (Fig. 1). The 24-h urinary calcium excretion was 546 mg (normal range 42–352). Ultrasound scans of the thyroid followed by parathyroid scan showed diffuse tracer accumulation in parathyroid topography.

## Treatment

**Figure 2:**
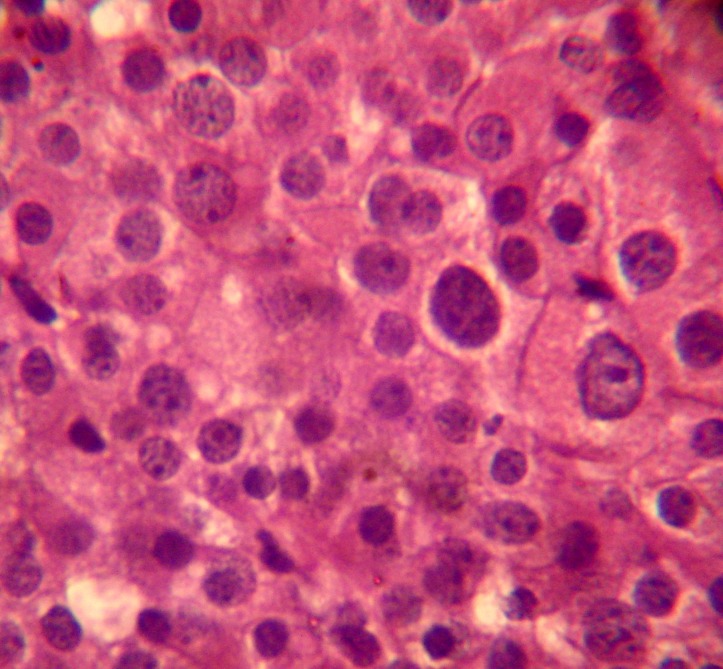
Histopathology of parathyroid showing adenomatous changes.

Subsequently, the patient was referred for parathyroid surgery. Histopathology of parathyroid glands revealed diffuse hyperplasia (Fig. 2).

## Outcome and follow-up

At last follow-up, he is doing well with neurological manifestations having largely subsided and so too the hypercalcaemia and parathyroid levels.

## Discussion

Diagnosis of MND is mainly based on some clinical criteria where there are different combinations of upper and lower motor neuron signs, which are commonly progressive in nature and all alternative explanations had been ruled out to a reasonable degree (3) (4) (5) (6). What this means in effect is that the clinician uses his clinical judgement as per the signs and symptoms and then looks for investigations such as electrophysiological studies, neuro-imaging and laboratory tests in order to prove or disprove that the patient is suffering from MND. The most commonly encountered form of MND is ALS, which is incurable and progresses relentlessly causing disability and eventually death (7). Although ALS is sporadic in occurrence, men are known to suffer from ALS twice more commonly than women with a peak between 55 and 75 years of age (7). Respiratory failure is the most common cause of death and patients are not expected to live for more than 3–5 years after the onset of symptoms (7). Most treatment is just supportive in nature. Although Riluzole has been approved for treatment of this condition, it has as yet not shown any survival benefit.

Noting the fact that MND is an untreatable disease condition, all efforts should be made to look for treatable/correctable conditions that are known to mimic MND such as magnetic resonance imaging spine to look for structural anomaly and cervical myelopathy, serum vitamin B12 level (for subacute combined degeneration), iPTH (for hyperparathyroidism), serum protein electrophoresis with immunofixation (for multiple myeloma and MGUS) and free thyroxine with TSH (for hyperthyroidism) (8).

There have been authors who have reported some correlation between hyperparathyroidism and MND, but no conclusive evidence has as yet been established. Similarly, there have been reports of MND/ALS with aberrant calcium metabolism, but till date no causal relationship has been established. Besides, surgical removal of parathyroid (as in the case of treatment for hyperparathyroidism due to parathyroid adenoma) has not been associated with improvement of MND (9). As shown by Jackson *et al*., (9) resection of parathyroid adenoma led to correction of the biochemical parameters but still patients with MND deteriorated and died within ∼3 years. A couple of decades ago, Patten & Mallette (10) reported that 50% of MND patients had radiological evidence of bone disease and 20% had abnormal serum calcium levels. They also postulated that ‘disturbances in calcium metabolism may stimulate MND and place patients with both primary and secondary hyperparathyroidism at risk for ALS’. However, it has now been proved that hypercalcaemia and thyroid dysfunctions themselves can present with signs and symptoms of MND and that hypercalcaemia is not a cause of MND (8) (11).

Patients with PHP can present with proximal muscle weakness, but this commonly affects the lower limbs and is symmetrical in presentation along with brisk deep tendon reflexes with down-going plantar response. Patients with hypercalcaemia may even present with features of bulbar paralysis and abnormality of tongue movement in addition to muscle cramps (12). However, there are some major clinical differences between the presentations of ALS and PHP, i.e. PHP often presents with loss of pain and vibration sense in the glove-and-stocking areas, ataxia, decreased arm swing, poor memory, disorientation, emotional liability, personality change, anxiety, disorientation and hallucination (12). Since the 19th century, there have been reports of muscle weakness with hyper-reflexia and muscle atrophy associated with PHP with improvement of symptoms after surgical removal of parathyroid adenoma (9) (13) (14) (15).

Our patient had presented with features of both upper and lower motor types of weakness with a normal sensory, autonomic and cerebellar functions. Hence, his clinical feature would lead any clinician to a diagnosis of MND. But his initial investigations and normal EMG–NCV forced us to rethink and exclude the MND mimickers. During investigation for MND mimickers, we found the serum calcium level to be highly raised with bilateral renal calculus. Further investigation established this hypercalcaemia to be due to PHP.

## Patient's perspective

Consent was obtained from the patient in the prescribed format (which has been uploaded separately).

## Patient consent

Consent was obtained from the patient before submission of this article.

## Author contribution statement

M Ghosh and K Ghosh contributed to prepare the original manuscript. A Bhattacharya, S Chakraborty and A Chatterjee helped in data collection and analysis, and S K Jatua has edited the final version.
